# Evidence for the Efficacy of Commercially Available Wearable Biofeedback Gait Devices: Consumer-Centered Review

**DOI:** 10.2196/40680

**Published:** 2023-04-19

**Authors:** Kedar K V Mate, Ahmed Abou-Sharkh, Maedeh Mansoubi, Aeshah Alosaimi, Helen Dawes, Wright Michael, Olivia Stanwood, Sarah Harding, Daniel Gorenko, Nancy E Mayo

**Affiliations:** 1 Faculty of Medicine and Health Sciences McGill University Montreal, QC Canada; 2 Medical School, University of Exeter Exeter United Kingdom; 3 King Faisal Specialized Hospital and Research Centre Riyadh Saudi Arabia

**Keywords:** wearables, technologies, walking training, rehabilitation, biofeedback, mobile phone

## Abstract

**Background:**

The number of wearable technological devices or sensors that are commercially available for gait training is increasing. These devices can fill a gap by extending therapy outside the clinical setting. This was shown to be important during the COVID-19 pandemic when people could not access one-on-one treatment. These devices vary widely in terms of mechanisms of therapeutic effect, as well as targeted gait parameters, availability, and strength of the evidence supporting the claims.

**Objective:**

This study aimed to create an inventory of devices targeting improvement in gait pattern and walking behavior and identify the strength of the evidence underlying the claims of effectiveness for devices that are commercially available to the public.

**Methods:**

As there is no systematic or reproducible way to identify gait training technologies available to the public, we used a pragmatic, iterative approach using both the gray and published literature. Four approaches were used: simple words, including some suggested by laypersons; devices endorsed by condition-specific organizations or charities; impairment-specific search terms; and systematic reviews. A findable list of technological devices targeting walking was extracted separately by 3 authors. For each device identified, the evidence for efficacy was extracted from material displayed on the websites, and full-text articles were obtained from the scientific databases PubMed, Ovid MEDLINE, Scopus, or Google Scholar. Additional information on the target population, mechanism of feedback, evidence for efficacy or effectiveness, and commercial availability was obtained from the published material or websites. A level of evidence was assigned to each study involving the device using the Oxford Centre for Evidence-Based Medicine classification. We also proposed reporting guidelines for the clinical appraisal of devices targeting movement and mobility.

**Results:**

The search strategy for this consumer-centered review yielded 17 biofeedback devices that claim to target gait quality improvement through various sensory feedback mechanisms. Of these 17 devices, 11 (65%) are commercially available, and 6 (35%) are at various stages of research and development. Of the 11 commercially available devices, 4 (36%) had findable evidence for efficacy potential supporting the claims. Most of these devices were targeted to people living with Parkinson disease. The reporting of key information about the devices was inconsistent; in addition, there was no summary of research findings in layperson’s language.

**Conclusions:**

The amount of information that is currently available to the general public to help them make an informed choice is insufficient, and, at times, the information presented is misleading. The evidence supporting the effectiveness does not cover all aspects of technology uptake. Commercially available technologies help to provide continuity of therapy outside the clinical setting, but there is a need to demonstrate effectiveness to support claims made by the technologies.

## Introduction

### Background

Gait is one of the most frequently assessed attributes in clinical settings because gait impairment is the hallmark of several different health conditions [1,2]. Prevalence of poor gait and gait-associated impairments is on the rise because people are living longer, with multimorbidity of chronic conditions such as obesity, diabetes, and arthritis, and because of a global increase in the proportion of neurological conditions [3-5]. Gait impairments and walking limitations from aging, disease, or injury increase the risk of falls, joint damage, and a sedentary lifestyle, leading to a vicious cycle toward further deterioration [6,7]. To illustrate, gait and balance impairments have been shown to increase with age—from 10% among individuals aged between 60 and 69 years to >60% in individuals aged >80 years [8]. Gait and mobility challenges are among the main concerns for older adults and people with neurological conditions and are a major reason for seeking rehabilitation services. There is considerable evidence to support the effectiveness of gait training in older adults and people with neurological conditions [9-15]. Although traditional therapist-led gait training strategies are well-accepted and effective in improving gait patterns, the effects abate with cessation of training [16,17]. Hence, traditional therapist-led gait training alone will not translate into sustained neuromuscular change nor lead to the behavioral change needed for physical activity guidelines to be met.

Effective and accessible treatments for gait impairments will increasingly be needed with the aging of the population and as people with health conditions live longer. Skilled therapy professionals are a limited resource, and therapy is rationed; furthermore, rehabilitation is a global target of the World Health Organization’s 2030 strategy, with key areas for action to increase affordable services and use technology to address this need to assess and reassess how individuals mobilize and move and implement long-term training programs [18]. Increasingly, people with gait vulnerabilities and their family members will turn to technological solutions to supplement and extend rehabilitation services [19,20]. Technological innovations are poised to close the gap between demand and supply [21]. There is no doubt that older adults and people living with health conditions would benefit from focused gait training beyond what is offered during a clinical visit [22,23]. Technology can provide people with opportunities to practice gait-related skills outside the clinical environment and gain *ownership* over their therapy [24]. There is evidence to support that technology alone can influence positive behavior and that smartphone apps have been shown to reduce sedentary time by 41 minutes per day [25,26]. These effects are thought to be a result of the user’s ability to self-monitor and self-correct, thus providing them with more control and responsibility for their own therapy [26]. Given the unmet need for access to rehabilitation services and the need to continue therapy outside clinical settings, the commercialization of technology is timely and necessary.

Available devices range in sophistication from nonelectronic shoe insoles and walking aids to inertial or pressure sensors. Most of the technologies used have gait assessment functionality, but there is now increasing interest in harnessing the capacity of wearable sensors for providing biofeedback. The literature is rich in supporting the effectiveness of biofeedback in improving gait patterns in healthy and clinical populations [27-30].

There is an increasing number of devices that claim to improve gait impairments through biofeedback. However, it is still rare for these devices to be available to the consumer; most are still tied to a laboratory setting. There is an urgent need to move technological innovations from research laboratories to the people who would benefit the most—those with gait impairments. The COVID-19 pandemic has alerted us to the vulnerability of seniors and people living with chronic health conditions when they were no longer able to access clinical and community resources [31-37]. In addition, the growing size of the older population means that one-on-one treatment will no longer be feasible, and a self-management strategy facilitated by technologies will be needed [31-33,38].

The market of people needing gait training technologies is huge. As a result of direct access of the general public to several technologies, the impact of evidence presented on the websites could affect purchasing behavior. A study on the purchasing intention of consumers who shop on the web found that “high involvement” consumers, defined as people living with health conditions who need to improve their gait to meet functional demands or mobility needs and are intently looking to purchase something specific, were more likely to purchase a product if the number of quality reviews was high [39]. Individuals with gait impairments may be considered “high involvement” consumers and, therefore, may purchase related products based solely on available reviews that may or may not have evidenced research quality.

Gait training technologies must be appealing with features such as product attractiveness, functionality, and price, as well as be supported by robust research demonstrating usability, reliability, efficacy, and effectiveness. All these features are equally important; an attractive product that does not work or a product that does work but is expensive would not be appealing. Although the attractiveness of technologies is often featured on websites, the evidence for efficacy could be hard to locate. Furthermore, the public is not likely to have access, time, or training to find the scientific literature and critically appraise the content to guide the decision to purchase such products.

### Objectives

The objectives of this customer-centered review were to create an inventory of devices targeting improvement in gait pattern and walking behavior and identify the strength of the evidence underlying the claims of effectiveness for devices that are commercially available to the general public [40].

## Methods

### Pragmatic, Iterative Approach

**Figure 1:**
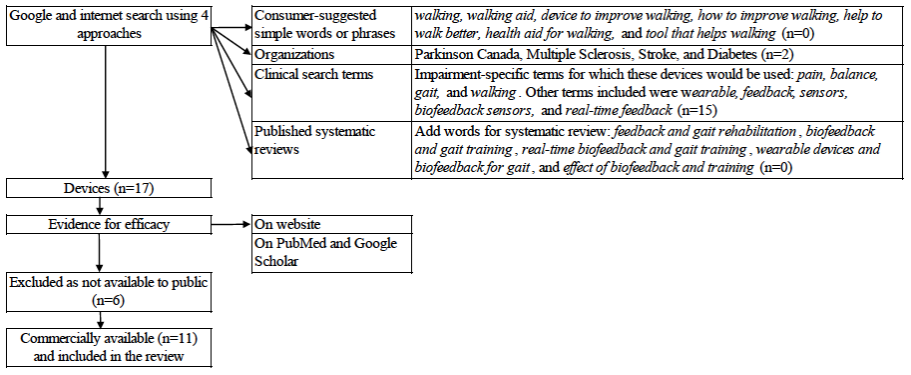
The steps taken to identify commercially available biofeedback devices to improve gait pattern and walking behavior.

As there is no systematic or reproducible way to identify technologies available to the public to help improve gait, we used a pragmatic and iterative approach. Our search strategy involved a search of gray literature as well as published literature. Figure 1 shows the 4 approaches used to identify a list of commercially available biofeedback devices. We used simple words, including those suggested by laypersons. Our focus was on devices that provided feedback, but this would not be thought of by the consumer. Therefore, we supplemented this strategy by searching for condition-specific organizations or charities because they might endorse such devices. This search yielded 2 feedback devices. We also performed a search using clinical impairment–specific search terms, and this yielded another 15 feedback devices. Finally, we searched for systematic reviews covering gait but found no new devices [41-45]. The search was first conducted in October 2021 and repeated in December 2022. Once we had a list of devices, we searched for evidence of efficacy published on the device web page as well as on PubMed and Google Scholar using the device name to search.

A findable list of technological devices targeted to health conditions was extracted separately by 3 authors and compared for completeness. For each device identified, the evidence for efficacy or effectiveness was extracted from material displayed on the websites, and full-text articles were obtained from the scientific databases PubMed, Ovid MEDLINE, Scopus, or Google Scholar. This step was carried out by MM, AA, MW, OS, SG, DG, and HD; any conflicts were resolved in consultation with KM and AA-S. Finally, KM and NEM organized the results into tables and reverified all data and assigned levels of evidence. A level of evidence was assigned to each study involving the device using the Oxford Centre for Evidence-Based Medicine scale [46,47].

### Levels of Evidence

The *levels of evidence* rating system is a method of quantifying the best clinical evidence that is available about the efficacy and safety of treatment approaches that are destined to be implemented in clinical care [46,47]. The Oxford Centre for Evidence-Based Medicine scale was used because it provides the best granularity of evidence arising from the majority of trials of new technologies that usually are not included in meta-analyses and do not have randomized clinical trials with large sample sizes producing narrow CIs. Multimedia Appendix 1 shows the Oxford Center for Evidence-Based Medicine levels of evidence.

Information on the target population, mechanism of feedback, evidence for efficacy or effectiveness, and commercial availability was obtained from the published material or websites. Only devices that claimed gait rehabilitation or gait quality improvement through any sensory feedback mode—visual, auditory, haptic (tactile or kinesthetic), or vibration—were included. The devices were excluded if the technology was not targeted to any health condition or if it targeted high-functioning populations such as athletes or healthy individuals. The term *feedback* is defined as a physiological or performance signal arising as a result of human movement that, in turn, generates an output (error or correct performance) that is relayed back to the user and that has the potential to modulate (enhance or diminish) subsequent movement.

## Results

### Overview

The search yielded 17 wearable devices that claimed to target improvement in gait quality through various types of feedback: 11 (65%) were commercially available, and 6 (35%) were at various stages of research and development. Of the 11 commercially available devices, 2 (18%) were sold under the trademark WalkWithPath: Path Finder Laser Shoes and Pathfeel. The inclusion of the devices was appraised by KM and NEM. Table 1 presents a brief description of the devices (grouped into insoles, wearable sensors, and vests or walking aids), feedback type, target condition, and components. The devices are organized according to availability: directly available to clients or only for research purposes and thus not commercially available. Among the 11 commercially available devices, there were 6 (55%) insoles, 4 (36%) wearable sensors, 1 (9%) vest, and 1 (9%) walking aid; for example, BalancePro insoles, which provide only passive sensory feedback, and FeetMe insoles, which have embedded sensors to provide different types of feedback, including electrical stimulation or an auditory signal. All technologies used a variety of biofeedback (positive, negative, and continuous) and offered options for choosing or providing a single preset sensory stimulus—auditory, haptic, visual, or vibration—enabling users to set individual preferences. A few of the devices offered practitioners and consumers a choice to select the feedback frequency and type of stimuli. Regarding choosing the type of sensory stimulus, there is no information available on the efficacy of one sensory stimulus compared with that of another. Most devices target gait improvement for people with neurological conditions, specifically people with Parkinson disease.

**Table 1:** Description of gait training devices (1) available to consumers and (2) for research only.

Gait rehabilitation devices			Feedback type		Condition		Interface
**Targeted: directly available to clients**							
	BalancePro: insoles with raised edges that provide passive sensory feedback on soles to enhance proprioception [48,49]	Haptic (continuous feedback)		Older adults, impaired circulation, and neuropathy		None	
	FeetMe Stimulate or Insole or Rehab: insoles with embedded sensors that collect gait and balance data and provide electrical stimulation at the foot or ankle to correct the gait pattern [50,51]	Auditory +^a^, haptic +, and visual +		Neurological conditions, obesity, chronic obstructive pulmonary disease, and older adults		Pressure and IMU^b^ sensors in insoles, electrodes, and Android app	
	WalkWithPath (Pathfeel): insoles with embedded pressure sensors that provide vibration corresponding to the pressure detected to enhance sensory information coming from the foot [52,53]	Haptic vibration (continuous feedback)		Parkinson disease and peripheral neuropathy		Pressure sensors and Bluetooth connection to smartphone app	
	Vibrating Insoles (Wyss Institute): insoles that provide subthreshold vibration continuously to enhance natural sensory information coming from the foot to improve balance and step consistency [54,55]	Vibration (continuous feedback)		Recreational athletes, older adults, and neurological conditions		None	
	Voxx Human Performance Technology socks and insoles: socks or insoles with embedded tactile pattern under the ball of the foot that stimulates the neural system to encourage the brain into a state of homeostasis [56,57]	Tactile (continuous feedback)		Poor balance and fall risk		None	
	Walkasins: insoles attached to ankle unit that detect pressure under the foot and provide vibration just above the ankles to improve balance and gait [49]	Haptic vibration +		Asymmetric gait (stroke) and neuropathy		None	
	Heel2Toe: sensor worn over the shoe that provides real-time auditory feedback on making “good steps” in which the heel strikes first [58]	Auditory +		Parkinson disease and older adults		IMU sensor and Android app	
	MEDRhythms: 2 wearable sensors attached to each shoe that provide rhythmic auditory feedback based on gait parameters to improve gait [59,60]	Auditory +		Neurological conditions		Headphones, IMU sensor, and smartphone app	
	CuPiD/Gait Tutor: 3 wireless sensors that evaluate real-time quality of gait and provide vocal message to walk safely, effectively, and smoothly [61]	Auditory and visual –^c^		Parkinson disease		Smartphone, IMU sensor, and docking station	
	WalkWithPath (Path Finder Laser Shoes): lasers attached to shoes bilaterally activated by body weight on the stance foot emit a horizontal light line on the floor on the opposite side for user to step on or over [52,53]	Visual (continuous feedback)		Parkinson disease		None	
	ReMoD V5.0 Type 1: vest with attached sensors that detect postural deviations and provide electrical stimulation at the anterior shoulders to correct trunk position when the user deviates past the set threshold [62,63]	Electrical –		Stroke, scoliosis, poor posture, and sensory or vestibular dysfunction		None	
	Isowalk: self-propulsive cane that guides user’s step forward [64]	Haptic (continuous feedback)		Fall risk		None	
**Research only, not commercially available**							
	Artistic 2.0: insoles that detect asymmetries and use a smartphone app display, high or low tone beeps, or long or short vibrations at the ankle to encourage symmetry	Auditory, visual, and vibration –		Neurological conditions and amputations		Silicon insoles with force sensors, a microcontroller, Bluetooth, and Android app	
	Walk-Even: insoles detect uneven weight distribution and use a speaker on the waistband to signal to the user to change weight distribution (auditory cue), or nociceptive electric stimulation is given on the thigh of the unaffected leg to encourage faster movement of the paretic limb	Auditory – and nociceptive –		Asymmetric gait (stroke)		Hard wired	
	AmbuloSono: wearable sensor worn on the leg provides auditory feedback (music) once a preset threshold is reached; if steps are too small, the music will stop	Auditory +		Parkinson disease		IMU sensor, audio speaker, iPod Touch, and Bluetooth	
	CueStim: electrical stimulation unit with electrodes on the quadriceps or hamstrings that continuously ramp up and down to overcome shuffling and freezing of gait	Electrical (continuous feedback)		Parkinson disease		Electrostimulation device, Bluetooth, smartphone app, and electrodes	
	VibeForward: 2 vibratory tactors placed inside the user’s shoes, a small electronics box containing a battery and an IMU sensor strapped around the ankle, and Bluetooth connection to a smartphone app; when activated by a switch on the device or a remote, the tactors provide vibration cycling from the hind foot to the forefoot in synchrony with the user’s step; the smartphone app acts as a remote control for the vibration	Vibration –		Parkinson disease		Tactors, IMU sensor, Bluetooth, and smartphone app	
	Walk-Mate: wearable sensor that provides auditory feedback on foot-ground contact; used as a gait compensation device to promote consistent cadence and gait symmetry	Auditory –		Neurological conditions		IMU, computer, headphones, and hard wired	

^a^+: positive feedback.

^b^IMU: inertial measurement unit.

^c^–: negative feedback.

### Effectiveness of Gait Training Devices

Textbox 1 presents information on population, intervention, control, outcomes, time, training, results, usability, and level of evidence with study design. Of the 11 commercially available devices, 4 (36%) have published evidence of efficacy reported in 10 studies with sample sizes ranging from 6 to 40: CuPiD/Gait Tutor, BalancePRO, Heel2Toe, and WalkWithPath [58,65-67]; for example, the BalancePro insoles are plastic insoles with a raised ridge around the perimeter that provide continuous haptic feedback and are targeted to people with Parkinson disease and older adults. The insoles are available for direct purchase on the company website, and the design patent application is under review. The evidence supporting the BalancePro technology comes from 2 crossover study designs and 1 randomized controlled trial, all at level 2b of evidence using the Oxford Center for Evidence-Based Medicine scale.

Textbox 2 outlines some important areas that would help judge the usefulness of technologies targeting gait from the perspective of consumers. These areas emerged from this review because the needed information was either absent from the papers or inconsistently presented. The list of technology-relevant items presented in Textbox 2 would be applicable for inventors publishing in the scientific literature.

**Evidence supporting the effectiveness of gait training devices.**

BalancePro (studies: 3; level of evidence: 2b)

- Authors, year: Jenkins et al [66], 2009
  - Population: individuals with Parkinson disease, n=40: 16 women and 24 men; age-matched controls, n=40: 25 women and 15 men
  - Intervention: facilitatory shoe insole
  - Control: conventional flat insole
  - Outcome: spatiotemporal gait parameters measured using GAITRite mat and muscle activity measured using electromyography (in 20 people with Parkinson disease and 20 controls)
  - Time: concurrent trials
  - Training: 10 walking trials: 5 with facilitatory insoles and 5 with conventional insoles
  - Results: group effect on velocity, step length, and step length variability
  - Usability: not reported
  - Level of evidence, study design: 2b, crossover (website and PubMed)
- Authors, year: Maki et al [68], 1999
  - Population: older adults, n=14: 6 women and 8 men; 7 healthy controls
  - Intervention: modified insoles
  - Control: none
  - Outcome: center of mass displacement and stepping reactions using force plates
  - Time: concurrent trials
  - Experimental condition: multiple transient perturbations and continuous perturbations: 40 and 16, respectively, for older adults and 56 and 24, respectively, for controls
  - Results: facilitation reduced the number of forward step reactions to perturbations
  - Usability: not reported
  - Level of evidence, study design: 2b, crossover (website and PubMed)
- Authors, year: Perry et al [67], 2008
  - Population: older adults, n=40: 19 women and 21 men aged 65 to 75 years
  - Intervention: facilitatory insole
  - Control: conventional insole
  - Outcome: lateral displacement of center of mass in relation to base of support during single-support phase
  - Time: 12 weeks
  - Training: 12 trials on 4 uneven surfaces wearing each sole
  - Experimental: 12 weeks of wearing randomly assigned sole
  - Results: outcome effect for 2 of the 4 uneven surface conditions
  - Usability: lower fall rate in intervention (25% vs 45%); mild discomfort occurrences reported for 17 out of 240 wear-weeks; 17 out of 20 participants would continue wearing
  - Level of evidence, study design: 2b, randomized trial (website and PubMed)

Walk With Path (studies: 3; level of evidence: 2b)

- Authors, year: McCandless et al [69], 2016
  - Population: individuals with Parkinson disease, n=20: 14 men and 6 women; mean age 68 years; independently ambulatory indoors, with freezing of gait
  - Intervention: laser cane, sound metronome, vibrating metronome, and vibrating walking stick
  - Control: no cueing
  - Outcome: frequency of freezing of gait episodes over 3-meter walk, first step length, second step length, forward center of mass velocity, sideways center of mass velocity, number of forward and backward sways and number of sideways sways, and forward center of pressure velocity (meters per second) and side-to-side center of pressure velocity
  - Time: concurrent trials, 3 per device and 3 control (total: 15 trials per participant)
  - Training: none
  - Results: 12 out of 20 participants contributed 100 freezing and 91 nonfreezing trials; laser cane was most effective for freezing of gait and for movement strategies to reinitiate movement, whereas vibrating walking stick was second most effective; vibration metronome disrupt movement compared with the sound metronome at the same beat frequency
  - Usability: not reported
  - Level of evidence, study design: 2b, crossover (website and PubMed)
- Authors, year: Barthel et al [70], 2018
  - Population: individuals with Parkinson disease with freezing of gait, n=21: 5 women and 15 men
  - Intervention: visual cueing using laser shoes
  - Control: no cueing
  - Outcome: duration and number of freezing of gait episodes
  - Time: concurrent trials, 5 trials each during on medication and off medication periods
  - Training: (1) walking back and forth over 10 meters; (2) task 1 plus counting down from 100 in steps of 7 or 3; (3) turning on command while walking, including 180° and 360° right and left turns; (4) walking to pick up a cone at 7 meters and then back carrying the cone; and (5) walking around obstacles placed on the walkway
  - Results: cueing reduced the number of freezing of gait episodes, both off (45.9%) and on (37.7%) medication, reduced the percentage of time frozen during the off period by 56.5% (95% CI 32.5-85.8), and reduced the percentage of time frozen during the on by 51.4% (95% CI –41.8 to 91.5)
  - Usability: not reported
  - Level of evidence, study design: 2b, crossover (website and PubMed)
- Authors, year: Velik et al [71], 2012
  - Population: individuals with Parkinson disease with freezing of gait, n=7: 1 woman and 6 men
  - Intervention: 3 cueing conditions: no cue, visual cue on for 10 seconds whenever freezing occurred, and continuous visual cue
  - Control: no cues
  - Outcome: average duration and number of freezing episodes under 3 conditions
  - Time: concurrent trials
  - Training: 6 tasks to be performed: (1) standing up from a chair and getting a glass of water from the kitchen, (2) going with the glass of water to the bathroom and leaving it on the washbasin, (3) walking to the bedroom and picking up a clothes hanger from the cupboard, (4) carrying a clothes hanger to the washing room and leaving it there, (5) going back to the chair, and (6) performing tasks 1 to 5 in reverse order, starting with task 5
  - Results: continuous cueing: mean duration of freezing reduced by 51%, with 43% fewer freezing of gait episodes; on-demand cueing: mean duration of freezing reduced by 69%, with 9% fewer freezing of gait episodes
  - Usability: not reported
  - Level of evidence, study design: 2b, crossover (website and PubMed)

Heel2Toe (studies: 2; level of evidence: 2b)

- Authors, year: Mate et al [58], 2020
  Population: older frail and prefrail persons, n=6: 4 women and 2 men
  Intervention: supervised training with the Heel2Toe sensor, 5 sessions over 2 weeks
  Control: none
  Outcome: spatiotemporal gait parameters and system usability
  Time: immediate and posttest feedback; end of training without and with feedback
  Training: supervised gait training and walking practice with the Heel2Toe sensor providing feedback for good steps; prescription of 5 exercises, 1 per walking component
  Results: immediate and posttraining response: 5 of the 6 participants displayed meaningful changes in terms of good steps, angular velocity, and coefficient of variation, whereas 1 high-functioning person showed no change
  Usability: 38-item responses: 25/38 (66%) were at optimal levels, and 9/38 (24%) were at the poorest levels
  Level of evidence, study design: 2b, sequential pretest-posttest design (website and PubMed)
- Authors, year: Carvalho et al [72], 2020
  Population: individuals with Parkinson disease, n=6: 4 women and 2 men
  Intervention: supervised training with the Heel2Toe sensor, 5 sessions over 2 to 3 weeks
  Control: none
  Outcome: spatiotemporal gait parameters and system usability
  Time: immediate pretest and posttest feedback; end of training without and with feedback
  Training: supervised gait training and walking practice with the Heel2Toe sensor providing feedback for good steps; prescription of 8 mobility exercises
  Results: immediate and posttraining response: of the 6 participants, 3 displayed meaningful changes in terms of good steps, 4 improved on angular velocity, and 1 reduced coefficient of variation
  Usability: 24-item responses: 17/24 (71%) were at optimal levels, and 9/24 (37%) were at the poorest levels
  Level of evidence, study design: 2b, randomized clinical trial (website and PubMed)

CuPiD/Gait Tutor (studies: 2; level of evidence: 2b)

- Authors, year: Ginis et al [65], 2016
  Population: individuals with Parkinson disease, n=40: 8 women and 30 men independently ambulatory for at least 10 minutes, with freezing of gait
  Intervention: supervised weekly visits for 6 weeks plus recommendation to walk at least 3 times per week for 30 minutes with feedback and cues provided separately
  Control: walking training with no feedback
  Outcome: gait speed, stride length, and double support time for comfortable gait and dual-task gait conditions; balance evaluated using Mini Balance Evaluation Systems Test; Four Square Step Test; Falls Efficacy Scale-International; 2-minute walk test; freezing of gait; Unified Parkinson’s Disease Rating Scale, part III; cognition; and quality of life
  Time: pretest-posttest training (6 weeks) and retention (4 weeks)
  Training: weekly home visits for 6 weeks
  Results: single-task and dual-task gait speeds improved within group at posttest and follow-up assessments; intervention group improved on balance at posttraining assessment
  Usability: not reported
  Level of evidence, study design: 2b, randomized clinical trial (website and PubMed)
- Authors, year: Ginis et al [52], 2017
  Population: individuals with Parkinson disease, n=28: 5 women and 23 men; 14 age matched
  Intervention: 4 walks (continuous and intelligent cues, intelligent feedback, no information) over 6 weeks with at least 1 week between walks
  Control: no information
  Outcome: cadence, stride length, and fatigue
  Time: concurrent trials
  Training: comfortable 1-minute reference walk before testing
  Results: decrease in cadence in participants with Parkinson disease without cues or feedback; participants with Parkinson disease reported more fatigue with continuous cueing and intelligent feedback; increase in coefficient of variation in cadence in participants with Parkinson disease; and less variation in cadence with continuous and intelligent cueing in participants with Parkinson disease
  Usability: reported
  Level of evidence, study design: 2b, crossover (website and PubMed)

**Suggested content for reporting guidelines for the clinical appraisal of devices targeting movement and mobility.**

Problem to be addressed: gap that the technology is filling

- Functionality: assessment, treatment, or both
- Technology type: implant, robot, exoskeleton, biosensors, virtual or augmented reality, assisted living technologies, wearables, smart devices, trackers, remote monitoring, and chatbots
- Technology: describe in a way that it can be pictured without an image
- Level of technology: technology readiness level (levels 1 to 9) [48]
- Population: (1) health condition and special selection criteria and (2) level of technology readiness
- Technology: country-specific regulatory authority classification of the medical device; mechanism of action: actual or hypothesized; reliability of algorithm used in the technology; and comparability with existing methods: (1) comparing assessments: competing technologies and (2) comparing treatments: sham, nothing, usual care, alternative form of technology, alternative intervention, and attention control
- Experimental protocol: as per Consolidated Standards of Reporting Trials (CONSORT) or other reporting guidelines [50] or as per the Template for Intervention Description and Replication (TIDieR) and other reporting guidelines [56]
- Outcomes: biofunctional model linking the technology to proximal and distal outcomes:
  Proximal (explanatory) outcomes: technological metrics and impairment level from the patient’s perspective
  Primary (confirmatory) outcomes: clinically assessed activity outcomes (capacity)
  Distal (exploratory) outcomes: real-world assessed activity outcomes (performance) and health-related quality of life

Source of information: patient-reported outcomes, self-reported outcomes, performance outcomes, and technologically assessed outcomes [64]

- Results: as per CONSORT or other reporting guidelines; distributional parameters presented for every outcome, every time point, and every transition
- Safety: symptoms (new or aggravated), allergies, injuries, abrasions, and falls
- User experience: qualitative and quantitative information on positive and negative experiences with the technology; actions taken to remedy negative experiences
  Usability: quantitative measure of perceived usability
  Adoption: data on short-term update and data on long-term use
- Level of evidence: level of evidence classification system specified

## Discussion

### Principal Findings

This review identified a total of 17 wearable biofeedback devices targeting gait patterns and walking behavior. Of these 17 devices, 11 (65%) are commercially available to the public and have a dedicated website for direct purchase. Of these 11 devices, 4 (36%) had published evidence on effectiveness at level 2b according to the Oxford Centre for Evidence-Based Medicine scale (Textbox 1). There was no searchable evidence available for the efficacy or effectiveness of the feedback from the remaining gait training technologies (7/11, 64%). Evidence is primarily generated for 1 health condition, but the claims are generalized to other health conditions with similar gait impairments. There was limited to no data available on accuracy, reliability, usability, and safety. Almost all websites presented user reviews or testimonials, which are likely to be selective in favor of supporting the technology. It is important for clinicians to be aware that some scientific evidence supporting the technology may exist, but a consumer is most likely unable to access the published material. A consumer may be driven to purchase a device or not merely by reading reviews or testimonials.

This review provides a summary of commercial wearable gait training technologies that are currently available in the market or the development phase. A unique feature of this review is that it was conducted from a consumer’s perspective and then augmented by summarizing the evidence from scientific publications. Although the strength of the evidence supporting the effectiveness of these technologies is low or moderate at best, the claims on the website often outweigh the evidence. The results of our review can also be used by professionals involved in gait rehabilitation to direct their clients to promising technologies based on available evidence. These technologies can also be incorporated into treatment plans.

### Comparison With Prior Work

Several of the papers (9/10, 90%) that contributed evidence toward the efficacy and effectiveness of the wearable sensors failed to capture or report patient-centered outcomes or declared level of evidence. In summary, the quality of evidence was low. Only 36% (4/11) of the devices had searchable evidence for efficacy potential, with all studies being small-sample sized (Textbox 1). This calls into question the strength of the evidence and the generalizability of the findings outside the study population. Although the mechanism of action and information on spatiotemporal gait parameters have been reported for all devices, it is important to provide information on walking speed, distance, physical function, and walking behaviors such as step count or walking bouts. Overall, the approach to statistical analysis is rudimentary, and inference is mainly based on within-group *P* values rather than CIs. Lack of raw data in the published manuscripts, such as mean, median, SD, and range, prevented a calculation of between-group effects, effect sizes, and reliable change among other metrics that can potentially provide more interpretable information. Sample sizes are typically small, leading to a high degree of uncertainty in the results. Very few papers (4/10, 40%) reported information on missing data or steps taken to account for missing data and the potential impact on the conclusions.

### Strengths and Limitations

There is a challenge in searching for information on technology. A 2022 review evaluated the type and quality of information available on the web for aquatic physiotherapy targeted to people with Parkinson diseases [59]. The authors used a commercial *social listening* service Awario that searches social media platforms (eg, Twitter and Instagram) and the web for investigator-selected keywords [59]. The strategy used here was a form of *snowball* sampling where systematic reviews served as the source, and the web was searched for any devices named in these reviews.

Many commercial technology companies reported ≥1 clinical trials that are underway; yet, there is a lack of trial-specific information. A potential consumer is unlikely to track these details. It is important to consider the transparency and accessibility of scientific evidence when making evidence-based recommendations to consumers. There is limited research in this area, specifically from a consumer’s perspective. Although there is a need to provide therapy outside clinical settings, it is critical that companies marketing technologies do not *scam* people into buying products that are possibly noneffective or even harmful and that clear reporting standards for consumers are made mandatory for these technologies, similar to those now standard for food.

The approach taken here may not have yielded complete results, and, because new technologies are continually developed and added to or removed from the market, the results can quickly become out of date. Many technologies are developed in research settings and are not given a proprietary name until there is evidence to support commercialization. Hence, searching for earlier information is impossible. In addition, the inventors, the authors of the papers, and the entrepreneurs commercializing the technology may not be the same people; hence, an author search will also be fruitless. CuPiD/Gait Tutor is an example of a name change [61,65]. Finally, there is no *gold standard* for rigorous, systematic gray literature search methods, and there are few resources on how to conduct this type of search; for example, the Cochrane Handbook, often cited as the gold standard for conducting systematic reviews, provides limited guidance and specificity for gray literature search methods [62]. In addition, the reporting of gray literature search methods in systematic reviews is often not held to the same high standards in transparency and reproducibility as the academic database search methods.

Therefore, the findings of this review are only valid based on the search conducted at the time. Given the difficulty in searching for gait training technologies, the search method reported here may be difficult to reproduce. Nevertheless, the information presented on the technologies discovered in this search uncovers existing gaps in the evidence and the reporting.

### Future Directions

As newer technologies for gait training are continually developed, it is important that the evidence supporting their efficacy and effectiveness is quickly made available to people to make an informed choice. Often, the published literature is unavailable to the general population because of journal paywalls. There was also a lack of consistency in reporting information related to usability, safety, or user feedback. Standards for reporting on research involving technological devices, in the form of reporting guidelines, seem to be a critical need to ensure that the data needed by the potential consumer are communicated.

There are several reviews on the efficacy and effectiveness of gait training technologies. One objective of the research is to build capacity and empower patients who wish to take charge of their health. By equipping people living with gait impairments with the opportunity to improve walking outside clinical settings through biofeedback is a step in the right direction, given the limited access to rehabilitation services. A few technological innovations were initiated along the commercialization path but were abandoned at different stages. Despite the many benefits of at-home therapy, some challenges exist, including device maintenance, battery life, and technological literacy. One study suggests that the most effective devices are those that have a “user-centered design,” meaning patients or practitioners are involved in the design process [30]. Many of the devices included in this review use this approach by consulting patients for feedback on comfort, ease of use, and preferred feedback modes, when applicable, during pilot studies.

Although there is strong interest from academic institutions and government agencies to transfer technologies from laboratories to clients, there is a need for due diligence on the part of both the institutions and industry to accurately report all the findings that not only support the science but will also influence a client’s or an organization’s decision to purchase the technology.

Research in the field of technology development seems to lack the rigorous research method standards required for drug testing, allowing some devices to enter the market based mainly on safety rather than efficacy. Almost all commercial devices overclaim the efficacy of the technology to other populations not supported by their research; for instance, a website will claim effectiveness for people with gait impairments when the device was tested only in people with Parkinson disease. Finally, it would truly benefit the general public to have a summary of the research in layperson’s terms similar to food standards.

The field of technology evaluation would benefit from reporting guidelines to extend the guidelines for reporting on randomized clinical trials (eg, Consolidated Standards of Reporting Trials [CONSORT]), such as are available for many different types of experimental studies, including pilot and feasibility studies and crossover designs, all of which can be found on the Enhancing the Quality and Transparency of Health Research (EQUATOR) website [54]. There are also guidelines for reporting on the features of the intervention (Template for Intervention Description and Replication [TIDieR]), which would be helpful to fully understand the intervention protocol and encourage replication [56]. For technology, it would be useful to provide additional information on user experience using both closed- and open-ended formats to identify challenges that users encounter with the technology.

### Conclusions

This review is the first of its kind from a consumer’s perspective that critically appraises wearable biofeedback gait devices found on the internet, the literature available on the respective websites, and the strength of evidence supporting the claims. The review highlights the need for providing standardized reporting of device capabilities as information accessible to the public when marketing commercialized devices. This review provides the public and health care practitioners with a summary of information that can be used to choose wearable biofeedback gait technologies or decide not to adopt them. The review covers 17 wearable devices that provide 1 form of feedback to improve gait and outlines the mechanisms claimed to underlie gait improvement. There was no predominance for biofeedback type (positive, negative, or continuous). A variety of biofeedback modes have been used (auditory, visual, or haptic), with auditory and vibratory haptic being the most common. The strength of the evidence supporting these devices from scientific sources was at 2a (lower randomized controlled trial) or 2b (prospective controlled trial—not randomized) level. Gaps in reporting all needed information for the consumer were uncovered. The propensity of small trials and heterogeneity of studies and conditions highlight the requirement for standardizing reporting of feedback intervention measures and doses to enable meta-analyses to move gait technological rehabilitation forward. Of note, there is a lack of evidence for motor learning interventions even in the field of sport, with a need for current evidence to be extended by theory-driven, high-quality studies to allow for more consolidated and evidence-based recommendations. Technology has the potential to advance the rehabilitation space and enable a better understanding of optimal interventions for learning and maintaining skills. Taken together, our findings target the need for clear reporting standards for gait interventions.

## Appendix

Multimedia Appendix 1 Oxford Centre for Evidence-Based Medicine levels of evidence. https://www.cebm.ox.ac.uk/resources/levels-of-evidence/ oxford-centre-for-evidence-based-medicine-levels-of-evidence-march-2009.

## Abbreviations

CONSORT: Consolidated Standards of Reporting Trials
EQUATOR: Enhancing the Quality and Transparency of Health Research
TIDieR: Template for Intervention Description and Replication
