# Supplementary figures and images for: Evidence for the Efficacy of Commercially Available Wearable Biofeedback Gait Devices: Consumer-Centered Review
Source: JMIR Rehabil Assist Technol. 2023 Apr 19;10:e40680. doi: 10.2196/40680 (PMC10157455; doi:10.2196/40680)

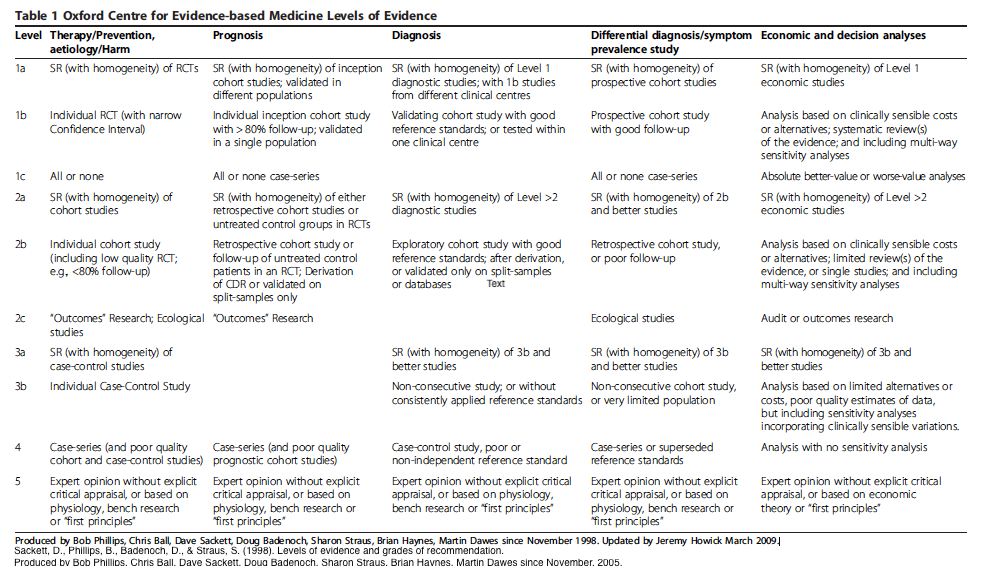

Supplement: Multimedia Appendix 1 [file rehab_v10i1e40680_app1.png]
